# A two-way rectification method for identifying differentially expressed genes by maximizing the co-function relationship

**DOI:** 10.1186/s12864-021-07772-2

**Published:** 2021-06-25

**Authors:** Bolin Chen, Li Gao, Xuequn Shang

**Affiliations:** 1 School of Computer Science, Northwestern Polytechnical University, 127 Youyi west road, Xi’an, 710072 China; 2 School of Software, Northwestern Polytechnical University, 127 Youyi west road, Xi’an, 710072 China; 3 Key Laboratory of Big Data Storage and Management, Ministry of Industry and Information Technology, 127 Youyi west road, Xi’an, 710072 China; 4 Centre for Multidisciplinary Convergence Computing (CMCC), 127 Youyi west road, Xi’an, 710072 China; 5 National Engineering Laboratory for Integrated Aero-Space-Ground-Ocean Big Data Application Technology, 127 Youyi west road, Xi’an, 710072 China

**Keywords:** Differentially expressed genes, Two-way rectification method, Functional related genes

## Abstract

**Background:**

The identification of differentially expressed genes (DEGs) is an important task in many biological studies. The currently widely used methods often calculate a score for each gene by estimating the significance level in terms of the differential expression. However, biological experiments often have only three duplications, plus plenty of noises contain in gene expression datasets, which brings a great challenge to statistical analysis methods. Moreover, the abundance of gene expression levels are not evenly distributed. Thus, those low expressed genes are more easily to be detected by fold-change based methods, which may results in high false positives among the DEG list. Since phenotypical changes result from DEGs should be strongly related to several distinct cellular functions, a more robust method should be designed to increase the true positive rate of the functional related DEGs.

**Results:**

In this study, we propose a two-way rectification method for identifying DEGs by maximizing the co-function relationships between genes and their enriched cellular pathways. An iteration strategy is employed to sequentially narrow down the group of identified DEGs and their associated biological functions. Functional analyses reveal that the identified DEGs are well organized in the form of functional modules, and the enriched pathways are very significant with lower *p*-value and larger gene count.

**Conclusions:**

An integrative rectification method was proposed to identify key DEGs and their related functions simultaneously. The experimental validations demonstrate that the method has high interpretability and feasibility. It performs very well in terms of the identification of remarkable functional related genes.

## Background

High-throughput experiments make it possible to evaluate the expression levels for thousands of genes in biological samples [1]. Gene expression data can reflect the gene expression level of the sample to be analyzed under different experimental conditions. Detecting differentially expressed genes (DEGs) across different experiments conditions is an essential step and sometimes the major goal in the statistical analysis of expression data [2]. It helps to understand the function of genes when cells respond to different conditions [3]. In addition, detecting DEGs can be a pre-step for clustering gene expression profiles or testing gene set enrichments [4, 5].

Numerous methods have been developed for identifying DEGs. A typical approach based on fold change (FC) [6, 7] calculates a ratio of the average expression values between *control* and *test* samples, where the threshold of 2-fold are usually employed to select genes under or above the threshold as DEGs [8]. Since the biological experiments often have limited number of duplications, plus plenty of noises contained in gene expression datasets, which makes the detection of FC based methods a little bit arbitrary [9]. To overcome this, many statistical approaches such as *t-test*, significance analysis of microarrays (SAM), etc, then become popular by modeling the distributional properties of gene expression levels. The SAM [10] method imposes a restriction on the variability of the genes by adding a value to the denominator of the *t-statistic*, excluding the genes that do not change or with high *p*-value. Another popularly used method called Moderated *t-statistic* (ModT) [9, 11] uses a t-distribution with augmented degrees of freedom. It is the integration of linear model with empirical Bayes, aiming to obtain a *p*-value for each gene and choose a feasible false discovery rate (FDR). These methods can calculate a score for each gene, and each of them can result in a ranked list of genes in order of their estimated significance level [12]. The performance of the statistics based algorithms depends on the number of available duplications. If there are less number of duplications in biological experiments, it is difficult to assume the distribution of data from a statistical point of view. Although the above methods combine mean and sample variance with the availability of *p*-values in cope with the high level of noise of dataset, they ignore the interactions between genes and obtain a set of isolated genes in a biomolecular network that are easily enriched in many unrelated biological functions, which is hard to analyze those genes’ functions from a system biology point of view and may become less efficiency to detect DEGs.

There are also methods that attempt to take into account the interactions between genes to reduce the effects of uneven distribution of the dataset. An influential approach called Characteristic Direction (ChDir) [12] towards the resolution of relationships between genes to increase statistical power. It incorporates a regularization scheme to maximize the use of dimensional information in expression data. Besides, the method provides an intuitive visualization of differential expression in terms of one single direction, which facilitates our subsequent analysis. The Min-Edge [13] method proposed in our previous study takes the interactions between genes into consideration, and detects DEGs based on the evaluation of differentially expressed edges, which is very useful for finding key genes related to disease.

We believe that giving two experimental conditions, DEGs should be involved in a certain biological function and may lead to a certain disease or phenotypic changes. Besides, they should be enriched in several distinct functions, such as functional related cellular pathways, GO terms or some common cellular functions [14]. Moreover, these pathways or functions should strongly related to each other under the biological experiment conditions. Thus, in this study, we propose a novel two-way rectification method to narrow down a set of potential genes and their associated cellular functions iteratively. The method starts from a set of high-confidence genes detected by any existed method, then one-way rectification is performed to search for the enriched cellular functions by conducting the genes into pathway enrichment analysis. Thirdly, an opposite direction of rectification is performed to rectify the DEG list by adding the meaningful genes that strongly related to target functions and removing the useless genes that related to only isolated functions. After conducting several rounds of this two-way rectification process, a set of closely related DEGs and pathways could be narrowed down by maximizing their co-function relationships.

**Fig. 1:**
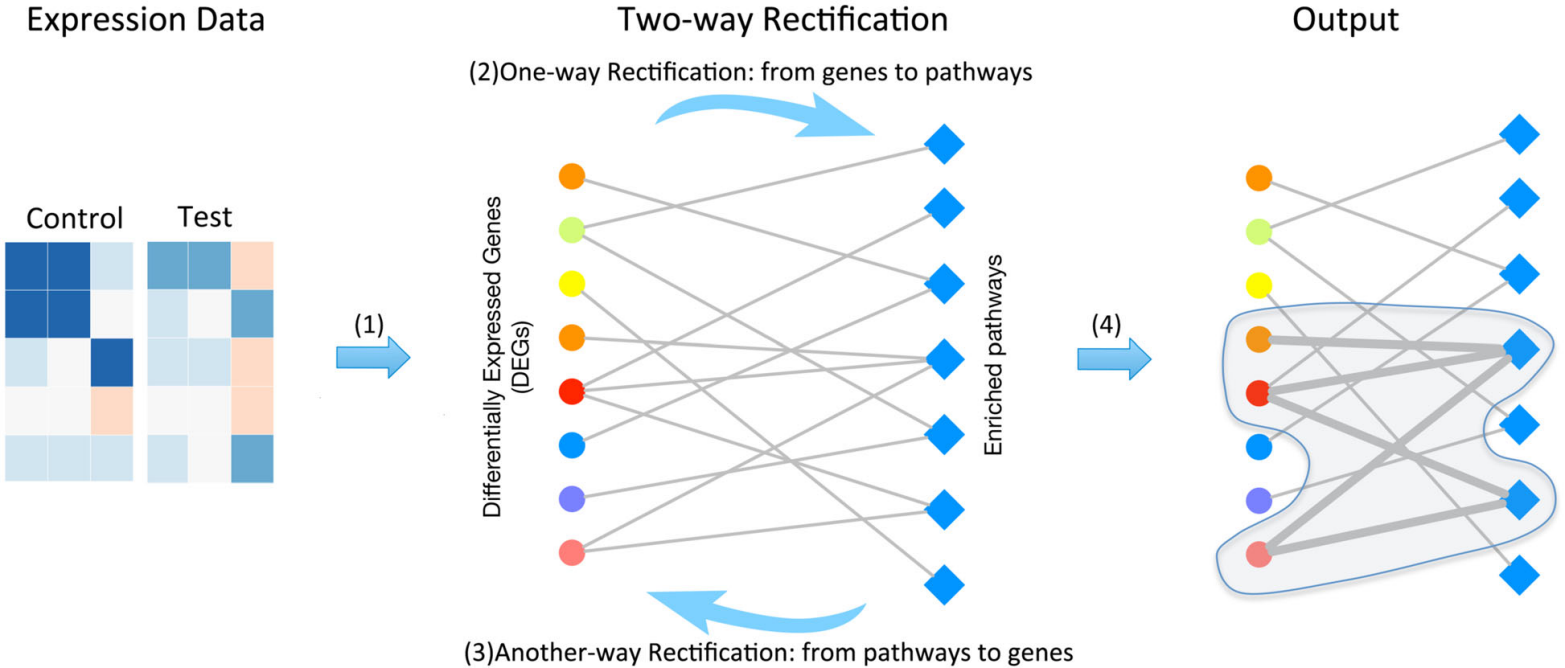
The principle of this two-way rectification. The color of expression data represent the different expression values of genes in *control* and *test* samples. The solid dot nodes represent a set of DEGs, and the diamond nodes with blue color represent those enriched pathways. The color of circle node depicts the differential expression level of the gene, the more significant the differential expression level of the gene, the closer the color of the node to red. Edge connects between a gene and a pathway means the gene is one of the annotated genes in that pathway. The thicker the edge, the closer the relationship between the gene and the pathway

The overall framework of the proposed two-way rectification method is illustrated in Fig. 1, where the cycle nodes represent a set of DEGs, and the diamond nodes represent the enriched pathways. Edge connects between a gene and a pathway means the gene is one of the annotated genes in that pathway.

## Results

The performance of the proposed method for finding DEGs from microarray datasets is evaluated using “Platinum Spike” and GSE41089 dataset. “Platinum Spike” dataset with ground truth information is used for the comparison of performance of proposed method with other gene selection methods. The microarray dataset with accession GSE41089 is used to validate the ability of designed method through biology point of view. The performance of the proposed method is further applied and compared with various gene detection algorithms, such as the ModT and Min-Edge.

The data of four groups (ModT, Two-way rectification using ModT, Min-Edge, Two-way rectification using Min-Edge) are divided into two comparisons (ModT vs Two-way rectification using ModT, Min-Edge vs Two-way rectification using Min-Edge), where Two-way rectification using ModT means the initial genes of Two-way rectification is selected from ModT. Similarly, the Two-way rectification using Min-Edge means the initial genes of Two-way rectification is selected from Min-Edge.

### The “Platinum Spike” dataset

#### Sensitivity verification of the parameters

In this section, we construct PPI networks based on the detected DEGs under different parameters of *n*, *m* and *i*. The ratio of the number of genes contained in the largest connected component in the PPI network to the number of DEGs detected at the *i*^*th*^ round of rectification is used as the connectivity of the network. The connectivity of the network reflects whether these detected genes are contribute to the necessary biological processes.

**Fig. 2:**
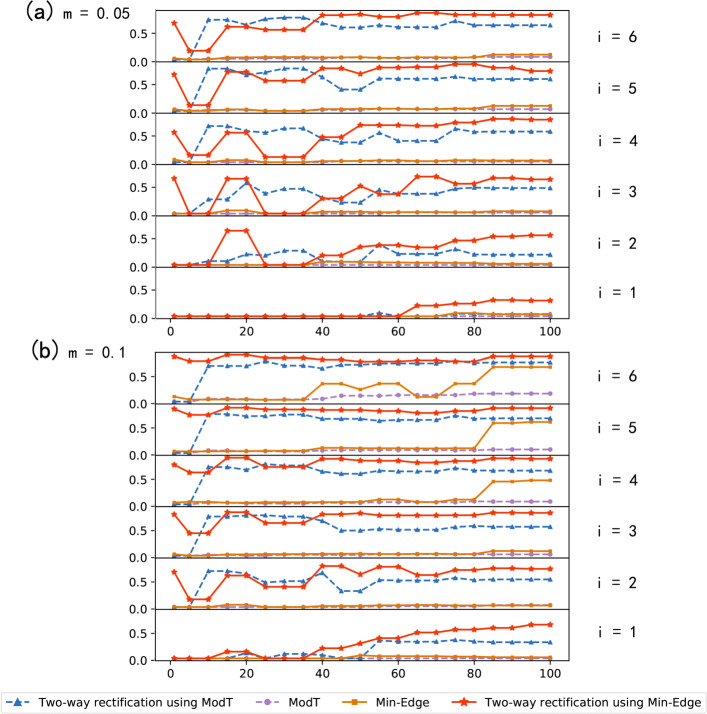
The connectivity of PPI networks constructed by DEGs at the *i*^*th*^ round of rectification under different parameters. The PPI connectivity is the ratio of the number of nodes in the largest connected component of the PPI network to the number of DEGs generated at the *i*^*th*^ round of rectification. The x-axis represents the number of initial gene list of the Two-way rectification, while the y-axis represents the connnectivity of PPI network constructed by DEGs obtained at the *i*^*th*^ round of rectification. **a** shows the connectivity results of each method when *m*=0.05, *n* ∈ (1, 100). **b** depicts the connectivity results of each method when *m*=0.1 and *n* ∈ (1, 100)

We execute the algorithm with *n* from 1 to 100, *m* from 0.01 to 0.1. Because of each values of parameters correspond to a sub-figure, so we give part of the results in this paper, results of *m* = 0.05 and *m* = 0.1 under different values of *n*. Figure 2 shows the changes of the connectivity of the PPI network constructed by DEGs found by each method under the corresponding parameters. From Fig. 2a, for the first (*i*=1) round of rectification with *m*=0.05, we can see that when select the a smaller number of candidate genes from initial gene list, even no genes have PPI correlations of each method. For the second (*i*=2) round of rectification, we can conclude that the connectivity of network obtained by Two-way rectification using Min-Edge is higher than Min-Edge first, but the connectivity of the network decreased with the increasing number of DEGs, which is due to the genes detected by Two-way rectification using Min-Edge contribute to some functional pathways but the centrality of genes in PPI network is small. Besides, it depicts the algorithm is not stable enough with smaller *n*. Moreover, the connectivity of the network is well performed when *n* ∈ (20, 100), which reflects the genes obtained by Two-way rectification based method are strongly interact with each other. When the number of initial genes *n* ∈ (80, 100), the algorithm obtained a lot of genes, these genes may co-expressed in the biological mechanisms because of the good influence on the connectivity of network. Considering the sensitivity of this convergence algorithm, we suggest that the values of *n* should be in (20, 80).

Figure 2b shows the connectivity of network with *m*=0.1. Similarly, we can find that at the *i*^*th*^ round of rectification, the Two-way rectification using Min-Edge performs well than Min-Edge, the Two-way rectification using ModT performs well than ModT. Comparing the Fig. 2a with b, more interacted genes can be detected when the values of *m* is 0.1 than 0.05.

#### Accuracy validation of the methods

The gene selection methods are employed for finding the DEGs based on differential expression between two experimental groups. And these two experimental groups are compared in terms of the AUC (area under the receiver operating characteristic (ROC) curve) scores. Algorithm with the highest AUC score performs the best. We computed true positive rate (TPR) as the number of true DEGs, true positive over the 1690 ground-truth DEGs, and the number of false positive gene (FPR) over the 11234 genes that are not differentially expressed, over all of the 12924 genes.

**Fig. 3:**
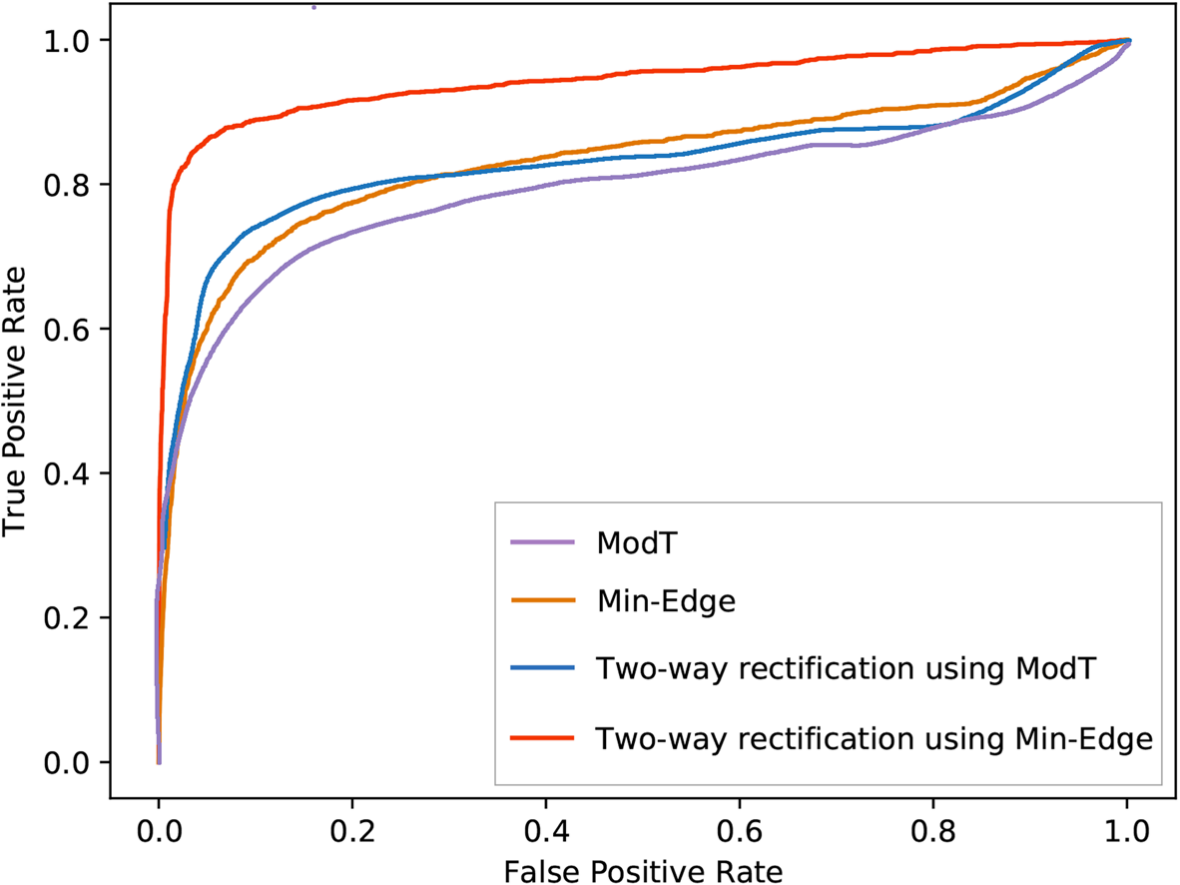
ROC curves of the four gene selection methods on the dataset of Platinum Spike. The red, orange, blue and purple lines depict the ROC curves of Two-way rectification using Min-Edge, Min-Edge, Two-way rectification using ModT and ModT, respectively. The AUC value of Two-way rectification using Min-Edge is 0.943028, which is higher than that of the other three algorithms

Figure 3 shows the results of the ROC curves and the corresponding AUC scores of Two-way rectification using Min-Edge, Min-Edge, Two-way rectification using ModT and ModT, respectively. According to the figure, these four algorithms achieved AUC scores of 0.943028, 0.842122, 0.839329 and 0.801652, respectively, among which the AUC score of Two-way rectification using Min-Edge is higher than Min-Edge, and the AUC score of Two-way rectification using ModT is higher than the competing algorithm ModT.

### The dataset of mice infected with T. cruzi

#### The number of identified DEGs

By conducting the ModT and Min-Edge algorithm on 8088 genes, 1565 and 1864 ranked DEGs are detected as the candidate genes, respectively. Based on these data, we select the top *n* DEGs from each method and regard them as the start gene list of the two-way rectification method. We execute the algorithm on the dataset with different values of *m* and *n*. As is illustrated in previous section, we choose the result of middle value with *n*=20,*n*=50,*m*=0.05,*m*=0.1, *i* ∈ (1, 9) as display in Table 1. When we select the top 20 ranked genes detected by ModT method as the initial gene set of two-way rectification, we obtain 38 DEGs after the first (*i*=1) round of rectification by using *m*=0.05 while 84 genes are obtained after the second (*i*=2) round of rectification. We can conclude that the number of DEGs be larger as the algorithm iterates more rounds.

**Table 1:** DEGs detected by two-way rectification method through ModT and Min-Edge, respectively

Method	n	m	Two-way rectification							
			*i* = 1	*i* = 2	*i* = 3	*i* = 4	*i* = 5	*i* = 6	*i* = 7	*i* = 8
ModT	20	0.05	38	84	146	221	297	384	469	549
		0.1	69	167	316	487	650	802	944	1068
	50	0.05	68	119	195	272	353	441	522	603
		0.1	113	264	427	582	733	873	1003	1122
Min-Edge	20	0.05	54	129	215	305	393	469	554	637
		0.1	96	255	427	600	760	907	1043	1167
	50	0.05	91	174	266	355	435	522	598	676
		0.1	154	335	507	668	820	961	1091	1213

As is clearly shown in Table 1, the number of DEGs is proportional to the selection of *n*, and *m* at the *i*^*th*^ round of rectification. The larger the value of *n* and *m* we set, the larger the number of final DEG set will be obtained under the same conditions. When taking the same number of *n* genes from Min-Edge and ModT method, respectively, it is found that two-way rectification will get more genes from Min-Edge than from ModT after the *i*^*th*^ round of rectification, since two-way rectification approach based on the pathway which takes the interactions between genes into consideration and provides the ranked genes correlated with functions while ModT only provides the isolated ranked genes.

#### Connectivity of PPI networks constructed by DEGs

PPI networks based on the detected DEGs under the different parameters are constructed for systematically view of connectivity. The number of DEGs in each largest connected network reflects whether these remarkable genes are involved in necessary biological mechanisms [15].

According to the results shown in Table 1, we compare ModT (Min-Edge) with two-way rectification in terms of the same number of DEGs obtained at the *i*^*th*^ round of rectification. To be more specific, we select the top *n* = 20 ranked genes from ModT method as the initial gene set of two-way rectification and obtain 38 DEGs after the first round of rectification of the proposed approach with *m*=0.05. Then select the same number of 38 DEGs from ranked genes identified by ModT method for the comparison of the two methods. PPI network then be constructed based on these two 38 DEG lists, respectively. After removing the genes with smaller degree in the network, we extract the number of nodes in the largest connected component. As a result, 16 nodes among 38 DEGs are detected by the ModT method are involved in the largest connected component, whereas 34 nodes are found by two-way rectification. Similarly, we give the comparison of Min-Edge vs two-way rectification using Min-Edge in terms of the connectivity of the PPI networks at each round of rectification.

**Fig. 4:**
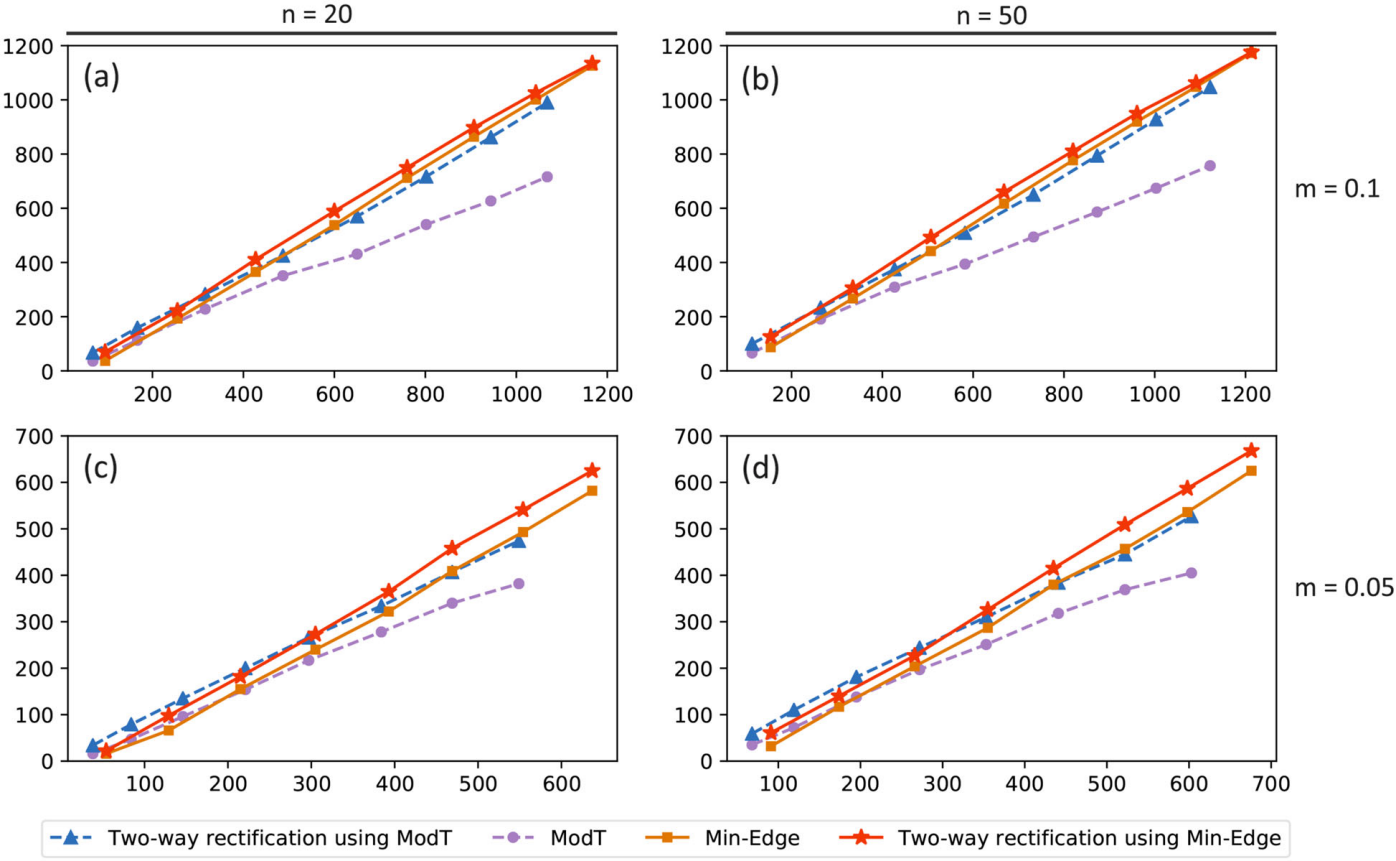
The connectivity of PPI networks constructed by DEGs at the *i*^*th*^ round of rectification. The data of four groups (ModT, Two-way rectification using ModT, Min-Edge, Two-way rectification using Min-Edge) are divided into two comparisons (ModT vs Two-way rectification using ModT, Min-Edge vs Two-way rectification using Min-Edge). The x-axis represents the number of DEGs generated at the *i*^*th*^ round (*i*=1, 2,..., 8) of rectification, while the y-axis represents the number of genes in the giant connected component of the PPI network constructed by these DEGs obtained through each round of rectification. The value of *n* represents the number of initial value of start genes of the two-way rectification method, while the *m* represents the ratio of DEGs that can be extended by the two-way rectification method at each round of rectification. **a** depicts the results at *m*=0.1 and *n*=20 between these methods. **b** depicts the results at *m*=0.1 and *n*=50 between these methods. **c** depicts the results at *m*=0.05 and *n*=20 of two comparisons. **d** depicts the results at *m*=0.05 and *n*=50 of two comparisons. Purple line: the result of the ModT method. Blue line: the result of the two-way rectification of which the start gene list is selected from ModT method. Red line: the result of the two-way rectification of which its initial gene list is selected from Min-Edge method. Yellow line: the result of the Min-Edge method

Figure 4a-d shows the comparison of connectivity of the largest component constructed by DEGs at each round of rectification between methods. Node in line means the number of genes in the largest connected network constructed by DEGs obtained at the *i*^*th*^ (*i*=1, 2,..., 8) round of rectification. When it comes to the same *m* visualized in Fig. 4c and d, at each round of rectification, the number of genes contained in the largest connected network when *n*=50 is larger than *n*=20 in each comparison. To be more specific, when *m*=0.05,*i*=1,*n*=20 shown in Fig. 4c, 16 nodes among 38 DEGs detected by the ModT method are involved in the largest connected component, whereas 34 nodes are found by two-way rectification. When *m*=0.05,*i*=1,*n*=50 shown in Fig. 4d, 32 genes among 68 DEGs detected by ModT are contained in the largest connected component, whereas 61 nodes are found by two-way rectification. Similarly as Fig. 4a and b in the case of *m*=0.1.

When taking the same *n*=20 genes as the start gene list of two-way rectification as Fig. 4a and c depict, the number of genes contained in the largest connected component obtained by each comparison at each round of rectification with *m*=0.05 is smaller than with *m*=0.1, which means the scale of the final genes list depends on the expansion factor *m*. Experiments show that the results of *m*=0.1 contain the most results of *m*=0.05.

When *m*=0.1,*n*=20 shown in Fig. 4a, we found that at each round of rectification, there are more genes in the largest component obtained by two-way rectification using ModT than by ModT, which depicts the PPI network obtained by two-way rectification is more connected than by ModT. Similarly, the PPI network is significantly connected in two-way rectification using Min-Edge compared to that in Min-Edge. The other three figures in Fig. 4 have same efficiency about this.

These results suggest that almost all the genes detected by two-way rectification method involve in the largest connected components of network, and reveal that these genes with closely correlations may play important roles in the activation of functional characteristics in mechanisms.

#### KEGG pathway enrichment analysis

Previous discussion confirms that the number of differentially expressed genes is proportional to the value of *n* and *m* in our method. In order to ensure the credibility and functional sensitivity of the differentially expressed genes, we give the results of taking different values for each parameter. In this section, we select the results when *i* = 6, *n* = 50 and *m*=0.1 as an example for subsequent analysis.

**Fig. 5:**
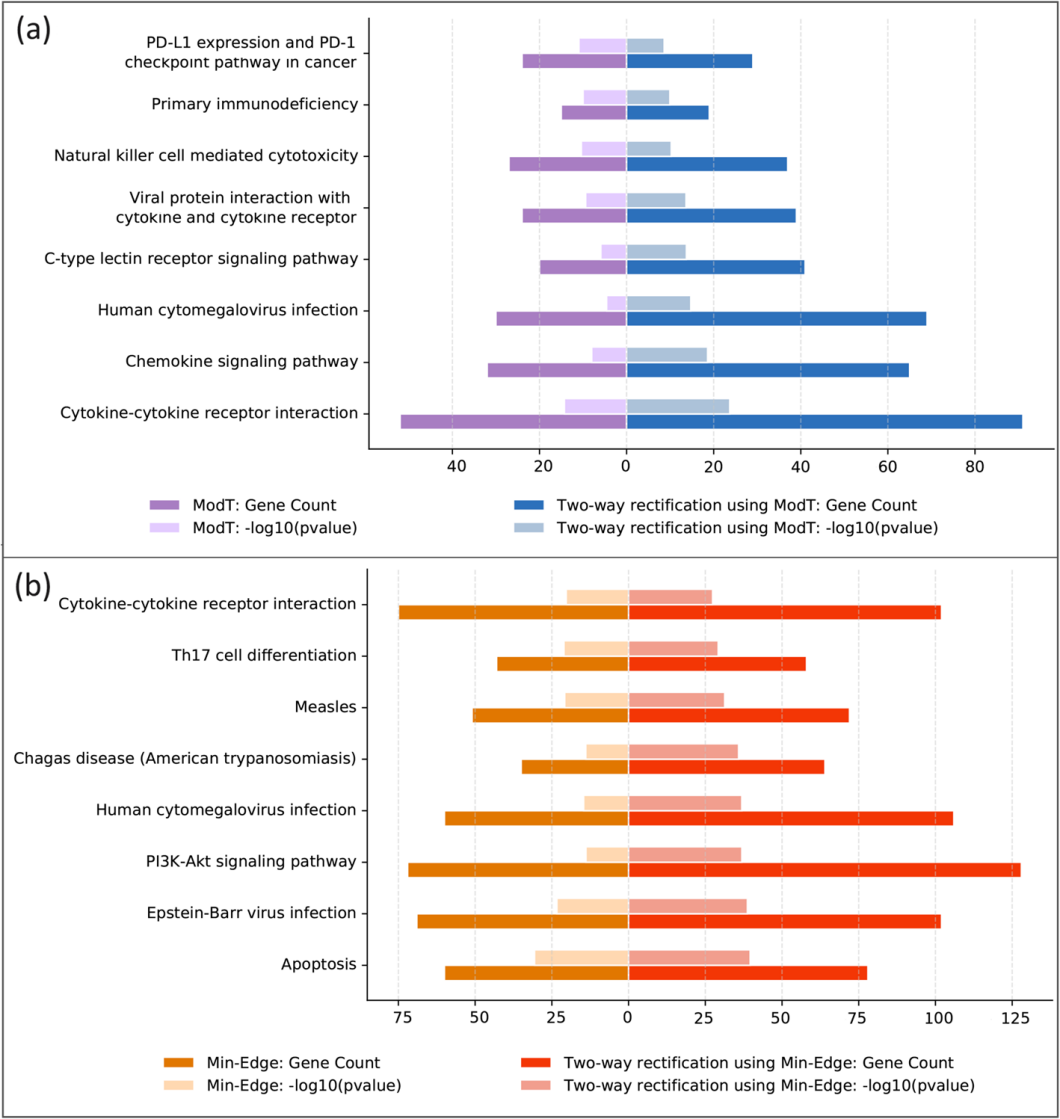
KEGG pathway enrichment analyses of DEGs. The dark color squares represent the number of annotated DEGs found in each pathway, while the light color squares represent the *p*-value of the pathway. **a** shows the comparison between the ModT method and the two-way rectification method using its DEGs. **b** depicts the comparison of the Min-Edge method and the two-way rectification method using Min-Edge

When *i* = 6, *n* = 50 and *m*=0.1, we obtain 873 DEGs by performing two-way rectification using ModT, while 961 DEGs are obtained by two-way rectification method when using the initial set of top 50 genes from the Min-Edge method. KEGG pathway analyses for the 873 (961) known DEGs from the two comparisons are performed, taking out the top 5 significant pathways of the ModT (Min-Edge) method and the two-way rectification using ModT (Min-Edge) respectively, and get 8 pathways after taking the combination for each comparison, as Fig. 5 shows. Figure 5a illustrates that for each enriched pathway, the *p*-value of which is smaller and it can annotate more DEGs by using the two-way rectification method, compared to that by using the ModT method. Figure 5b gives the comparison of DEGs between Min-Edge and two-way rectification method of which the start genes get from Min-Edge. For cytokine-cytokine receptor interaction pathway, there are more genes annotated and the pathway is more significant with smaller *p*-value enriched by two-way rectification than by ModT or Min-Edge. The results demonstrate that the pathways obtained by the two-way rectification method tend to be more significant with lower *p*-value and a large number of genes, among which are mainly likely to play important roles in the development of organisms.

## Discussions

***Strong associations between pathways and T. cruzi infection***

The gene expression dataset [16] we used in current study is generated to study the *Trypanosoma cruzi* infection on mice, with which the Chagas disease related pathways is strongly associated. We reviewed the literature and cated a catalog of some significant pathways including chemokine signaling pathway, cytokine-cytokine receptor interaction pathway, Toll-like receptor signaling pathway, NOD-like receptor signaling pathway and Chagas disease pathway, etc [16–19], which may contribute to pathogenesis of Chagas disease through the stimulation of proinflammatory cytokines and chemokines, leading to systemic alterations during the infection with *T. cruzi* parasites.

The DEGs detected by each method can enrich into the Chagas disease related pathways mentioned above. Table 2 shows the *p*-value corresponding to the pathway enriched by each method and the number of DEGs it annotates. We can see from the table that each method can enrich these pathways, among which the two-way rectification is more significant than the others, which demonstrate the genes we obtained by two-way rectification have high confidence in disease infection. Moreover, by using the two-way rectification, one can tend to enrich in a list of significant pathways with very low *p*-value and a large number of genes, such as cytokine-cytokine receptor interaction and Chagas disease pathway, which are strongly responsible to their real experiment conditions.

**Table 2:** Chagas disease related pathways

Pathways	ModT		Two-way rectification using ModT		Min-Edge		Two-way rectification using Min-Edge	
	*p*-value	count	*p*-value	count	*p*-value	count	*p*-value	count
Chagas disease (American trypanosomiasis)	1.88E-09	23	8.90E-14	38	1.16E-14	35	1.37E-36	64
Chemokine signaling pathway	9.88E-09	32	2.46E-19	65	5.01E-13	48	1.75E-31	85
TNF signaling pathway	3.02E-09	24	1.42E-13	40	7.69E-19	42	9.91E-31	62
T cell receptor signaling pathway	2.31E-09	23	1.83E-09	32	1.99E-11	31	1.10E-30	59
Cytokine-cytokine receptor interaction	5.17E-15	52	2.02E-24	91	4.91E-21	75	4.15E-28	102
Toll-like receptor signaling pathway	1.01E-09	23	5.94E-10	32	4.05E-15	35	9.82E-25	52
NOD-like receptor signaling pathway	4.22E-06	28	2.16E-06	43	8.53E-11	46	2.95E-16	66

**Fig. 6:**
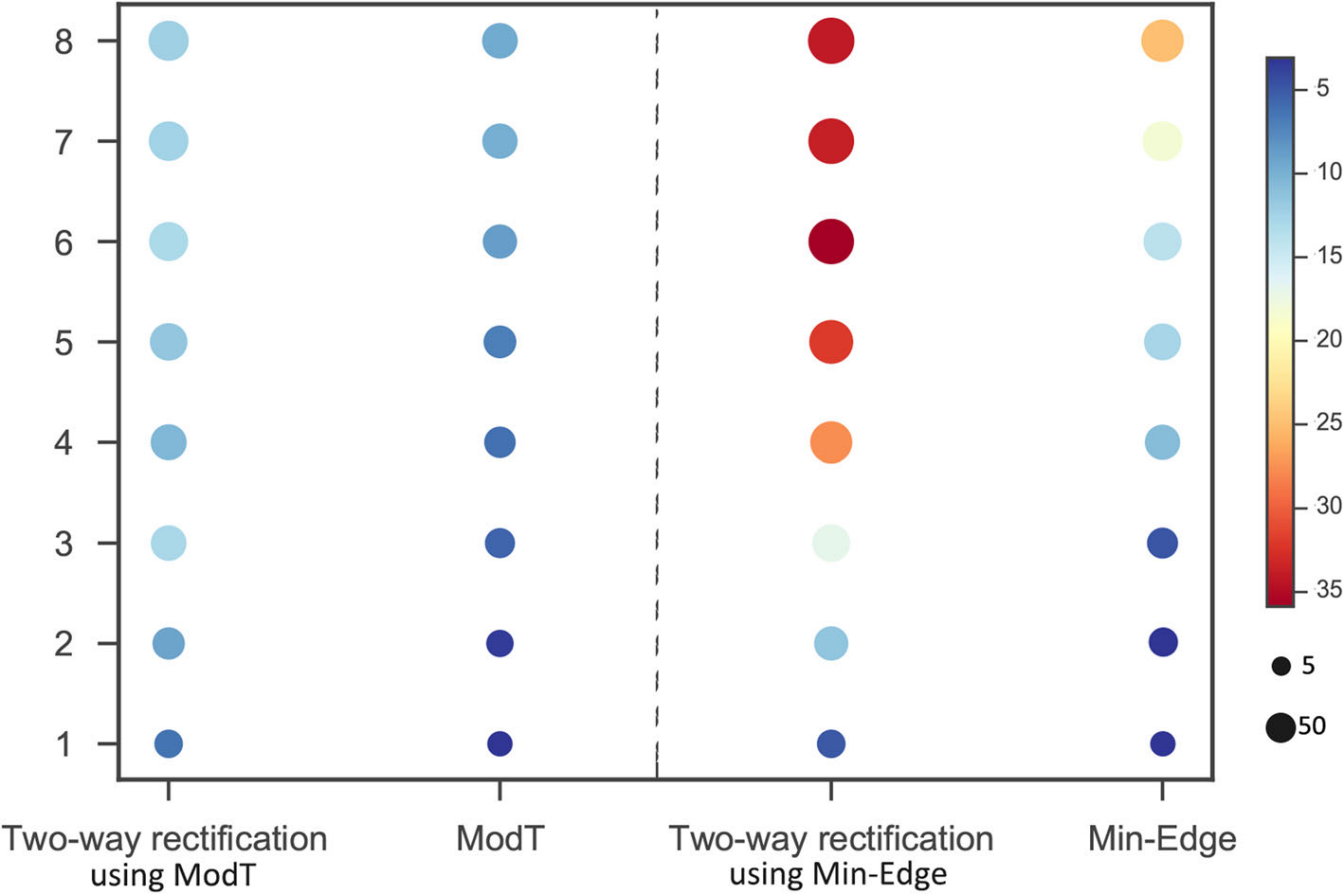
Chagas Disease pathway. The y-axis represents the times of execution of algorithm. Node represents the *Chagas disease* pathway acquired by each method at the *i*^*th*^ (*i*=1, 2,..., 8) round rectification, the size of which is proportional to the number of DEGs bearing it. The color of node denotes - *log*10(*p*-value) of the pathway enriched by each method, where red represent the most significant the pathway is

We thus extract the well-known Chagas disease pathway at each round of rectification for further observation. Figure 6 depicts that the two-way rectification of which initial gene list from ModT with lower *p*-value and more Chagas disease related genes compared with ModT at each round of rectification. When the initial gene list selected from Min-Edge, the two-way rectification also performs better than Min-Edge, which indicates that the genes obtained at the *i*^*th*^ (*i*=1, 2,..., 8) round of rectification are more concerntrated in Chagas disease. Besides, the two-way rectification method result in a significant level increase in the susceptibility to *T. cruzi* infection after the *i*^*th*^ round of rectification, revealing useful roles of DEGs detected by this way in againsting Chagas disease.

***Identification of guilted DEGs for pathways***

The number of DEGs obtained by two-way rectification using Min-Edge after the sixth rectification is 961 when *n*=50,*m*=0.1, among which 173 significant pathways are enriched. The interaction network between these pathways and DEGs is constructed to unravel the associations between key genes and functions by two-way rectification method of which the initial genes is obtained from Min-Edge. Edges in the network represent the genes contained in the corresponding pathways. The size of the node corresponds to the number of links, which reveals whether it is a significant pathway or functional related gene. After removing the genes with smaller degree, we obtained a network with 33 hub DEGs, 33 pathways and 497 edges.

**Fig. 7:**
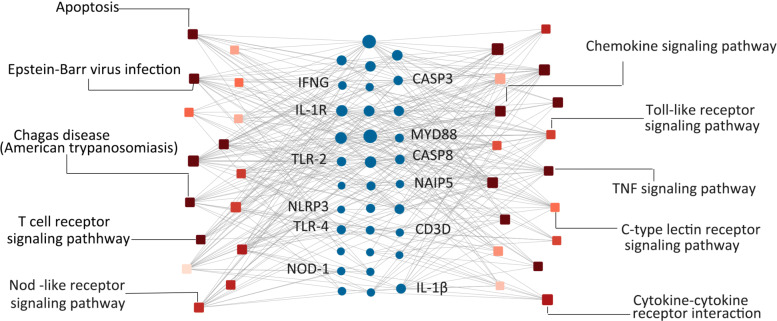
The association network between pathways and DEGs. Cycle nodes in the middle represents the detected DEGs, size of which corresponds to the number of links. Square nodes besides the genes are enriched pathways, the color of which denotes the *p*-value of the enriched pathway, the larger the square node, the more genes are annotated in this pathway

Figure 7 presents the associations between key pathways and DEGs detected by the proposed two-way rectification method. Each gene in the network has strong interactions with multiple cellular pathways, which indicates its potential probability as a driver gene of diseases due to its key role in the interaction network [20]. Previous studies have identified the significant genes of *T. cruzi* infection related pathways, such as TLR-2, TLR-4, TLR-7, IL-1 *β*, NAIP5, MYD88 and NOD1, and the genes of NLRs such as NLRP3, etc [16–19, 21], which have been recognized as the crucial for host resistance against *T. cruzi* infection by mechanisms. According to these known information, DEGs (CASP2, CASP8, NOD1, MYD88, TLRs, IL-1s, NAIPs) involved in Chagas disease here are screened, which have been proven to be correlated with the activation or inhibition of multiple Chagas disease related pathways [22].

As expected, these genes are well mapped into the Chagas disease related pathways, which reveals these identified genes have emerged as important components of these signaling pathways that account for detection of intracellular microbial infection. And DEGs obtained after the sixth rectification by two-way rectification method provides a list of significant genes which may competent at producing cytokines for the inflammasome in the host protection against *T. cruzi*.

## Conclusions

We have described a novel idea to identify DEGs by maximizing the co-functions between genes and pathways simultaneously. The method starts with known candidate genes and integrates pathway enrichment to obtain functional related genes. The basic assumption of the algorithm is the top ranked genes involved in pathways should play important roles in cellular functions.

To validate the feasibility of the proposed method, we use “Platinum Spike” dataset with ground truth information to compare the performance of proposed method with other gene selection methods, and the microarray dataset with accession GSE41089 is used to validate the ability and interpretability of designed method through biology piont of view. We identified the candidate genes and pathways related to Chagas disease for resisting *T. cruzi*, it is found that CASP2, CASP8, NOD1, MYD88, TLRs, IL-1s, NAIPs, etc, would be responsible for the *T. cruzi* infection. In addition, *T. cruzi* infection is regulated and mediated by genes related to chemokine signaling pathway, cytokine-cytokine receptor interaction pathway, Toll-like receptor signaling pathway, NOD-like receptor signaling pathway and Chagas disease pathway, etc. These findings establish the groundwork and imply that although the procedure relies on the genes that identified by existed methods, the proposed method outperforms other approaches in selecting functional related DEGs from microarray data, and it has high performance in either the number of DEGs enriched in pathways or the functionality of DEGs.

Given its excellent performance, we believe that the proposed method may shed new light on relevant biological mechanisms that would have remained undiscovered by the current methods. Further experiments will be focused on powerful larger samples with biological interpretation in identifying differentially expressed genes.

## Methods

### Data sources

The “Platinum Spike” dataset is downloaded from the National Center for Biotechnology Information Gene Expression Omnibus (GEO) website (accession GSE21344) [23] that consists of 18 spike-in samples (9 controls versus 9 tests). The designated FC associated file [24] contains 18952 probes, among which 1940 are known as differentially expressed probes. The robust multi-array average (RMA) method is used to normalize the probes. After data cleaning steps, we obtained 12924 genes, among which 1690 genes are known DEGs. The dataset can help us to validate the sensitivity of the proposed method and to evaluate the performance of different methods.

The second dataset is downloaded from the GEO website under the accession number GSE41089 [16]. This dataset contains 22,690 probe sets, 3 samples from uninfected mice (*control*), and 3 samples from infected mice (*test*). The probe-level data is analyzed using the MAS 5.0 [25] algorithm for the determination of the chip quality, including intensity value background correction, log2 transformation, and quantile normalization [26, 27], etc. The results of interest are confirmed through the robust R language *affy* Bioconductor package (*https://www.bioconductor.org/*). After these preprocessed steps, 8088 genes are retained for further analysis.

The pathway dataset of mouse and drosophila melanogaster is obtained from the database of KEGG. There are 317 pathways of mouse, and the total number of genes consisting of those pathways is 8578. Among them, 3492 genes are overlapped with the above 8088 genes. For drosophila melanogaster, there are 137 pathways and 5659 genes are involved in those pathways. Among them, 2383 genes are overlapped with the above 12924 genes.

The protein-protein interactions (PPIs) [28] dataset of the mouse and drosophila melanogaster is derived from the database of STRING [29] with a much larger number of associations. In this study, only PPIs between those 8088 genes and 12924 genes are selected to construct a PPI network and the score criterion is 0.4. After deleting the duplicated edges between the same pair of nodes and the edges connecting to itself, there are 7028 genes with 220817 edges for mouse and 11052 genes with 375793 edges left in this study.

### The two-way rectification method

Algorithm 1 gives the details of the presented DEGs identification method.


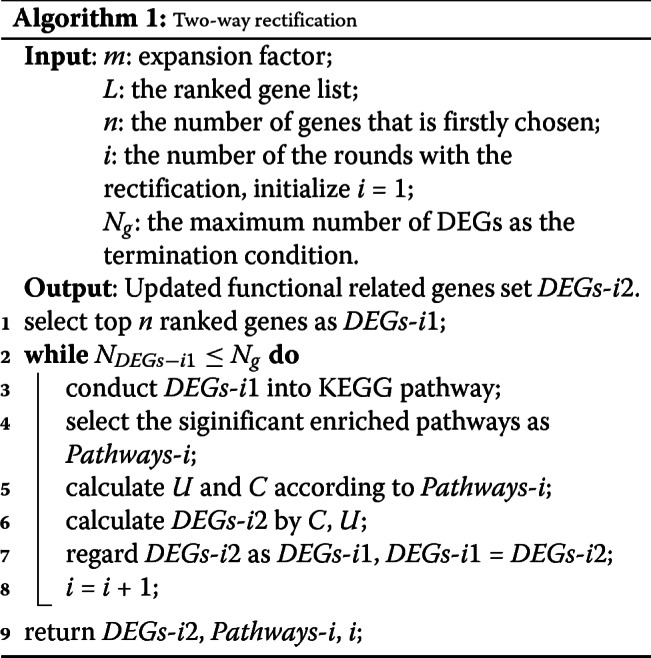


Giving two sets of *control* and *test* samples. Let *DEGs*-*ik* and *Pathway*-*i* represent different versions of the set of DEGs and pathways at the *i*^*th*^ round of rectification, respectively, where *k*=1,2. The gene expression of higher species is not only tissue-specific and development-stage specific, but also affected by environmental factors. The genes expressed in a single cell account for only 15% of the total number of genes. These expressed genes include the expression of newly emerging genes and the expression value of genes with different expression levels. That means the number of truth DEGs is less than 15% or even less. Thus, we suppose the number of DEGs should less than the 15 percent of the researching data, or even less. We proposed *N*_*g*_ as the 15 percentile of the number of the genes consist in researching data, which could more than the number of real DEGs.

The two-way rectification method is designed to narrow down a set of DEGs and their related cellular functions, the algorithm is summarized as follows and the Fig. 8 shows the details.

1. **The initial DEG list.** Since the proposed method relies on an existing DEGs identification approach, firstly we need to use an existing method (such as SAM) to calculate a differential score for each gene, and order these genes according to these differential scores and denote these ranked genes as *L*. For the input of the algorithm, the top *n* genes are selected as the initial candidate genes *DEGs*- *i*1, where *i* represents the number of rounds with rectification, *n* represents the number of DEGs that is firstly selected from all candidate genes.

**Fig. 8:**
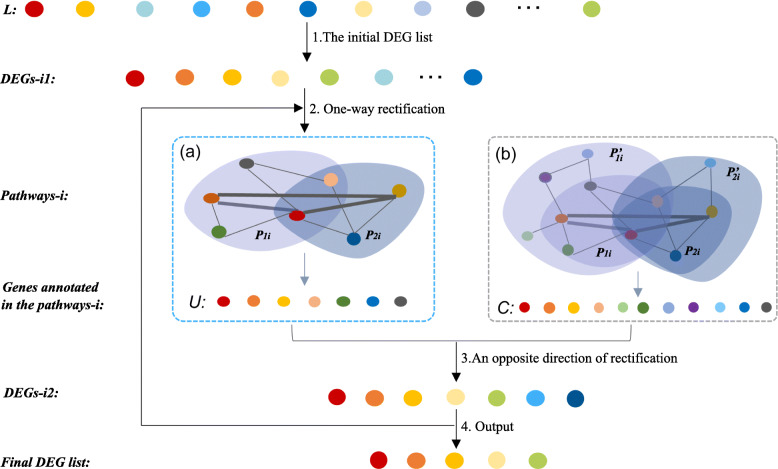
The details of the two-way rectification. The cycle nodes with color corresponding to the ranked genes that sorted according to the differential score, where the larger the differential score, the redder the color. *P*_*i*1_ or *P*_*i*2_ in **a** is a subset of $P_{i1}^{\prime }$ or $P_{i2}^{\prime }$ in **b**, cause only part of related genes will be found when conduct a gene list (such as *DEGs*- *i*1) into KEGG pathway enrichment, whereas $P_{i1}^{\prime }$ or $P_{i2}^{\prime }$ has more genes itself
2. **One-way rectification.** This step aims to obtain a set of high-confidence pathways accroding to the genes. As is shown in Fig. 8, for the *i*^*th*^ round of rectification, we use *DEGs*- *i*1 to conduct a KEGG pathway [30, 31] enrichment analysis and obtain a list of significant pathways *Pathways*-*i*. We found a subset of *DEGs*- *i*1 are annotated in *Pathways*-*i*, we select and order these genes as the set *U*. However, for each pathway in KEGG database, it includes but not only the genes in the set *DEGs*- *i*1, but also contains many annotated genes that are not shown in *P*_*i*1_ or *P*_*i*2_, so we rank these genes as set *C*.
3. **An opposite direction of rectification.** Once the *Pathways*-*i* is obtained, the *DEGs*- *i*1 list is updated by adding high confidence genes through the equation defined below,
  1 $$\begin{array}{@{}rcl@{}} D = m \ast (\vert C \vert \backslash \vert U \vert).  \end{array} $$
  Then the isolated functional related genes contained in *DEGs*- *i*1 and *U* are removed. The DEGs of *D* are obtained through the equation (1), where |*C*| and |*U*| represent the number of genes in *C* and *U*, respectively. The parameter *m* is a factor that controls the scale of expansion of *DEGs*- *i*1, which can help to reduce the noise arising from some weak functional related genes. It should be noticed that we aim to find genes that are strongly related to target functions. Hence, the value of *m* is usually very small. By doing this, the set of candidate functional hub genes is:
  2 $$\begin{array}{@{}rcl@{}} DEGs\text{-}i2 = U \cup D  \end{array} $$
  Thus, genes in the set of *DEGs*- *i*2 are regarded as new candidate function-related DEGs.
4. **Output.** Let *N*_*DEGs*-*i*2_ be the number of genes in the set of *DEGs*- *i*2. Let *DEGs*- *i*1 = *DEGs*- *i*2,*i*=*i*+1, repeat the step 2, 3, until *N*_*DEGs*-*i*1_≤*N*_*g*_. Through the above steps, we finally get an updated set *DEGs*- *i*2 and *Pathways*-*i*, where *DEGs*- *i*2 are strongly related to functions in *Pathways*-*i*.

## Data Availability

The datasets analysed during the current study are available in the GEO website (accession GSE21344 [23], GSE41089,[16]), and the PPI dataset from the STRING [29] database. The URLs of the datasets are as follows. https://www.ncbi.nlm.nih.gov/geo/query/acc.cgi?acc=GSE21344; https://www.ncbi.nlm.nih.gov/geo/query/acc.cgi?acc=GSE41089, and https://string-db.org/.

## Abbreviations

DEGs: differentially expressed genes
FC: Fold Change
SAM: significance analysis of microarrays
ModT: Moderated t-statistic
ChDir: Characteristic Direction
RMA: robust multi-array average
GEO: Gene Expression Omnibus
PPIs: protein-protein interactions
ROC: receiver operating characteristic
FDR: false discovery rate
FPRs: false positive genes
TPRs: true positive rates

## References

[1] Shaik JS, Yeasin M (2007). A unified framework for finding differentially expressed genes from microarray experiments. BMC Bioinformatics.

[2] Yu H, Pei D, Chen L, Zhou X, Zhu H (2017). Identification of key genes and molecular mechanisms associated with dedifferentiated liposarcoma based on bioinformatic methods. Oncotargets Ther.

[3] Aouiche C, Chen B, Shang X (2019). Predicting stage-specific cancer related genes and their dynamic modules by integrating multiple datasets. BMC Bioinformatics.

[4] Chen B, Yang M, Gao L, Jiang T, Shang X (2020). A functional network construction method to interpret the pathological process of colorectal cancer. Int J Data Min Bioinforma.

[5] Aouiche C, Chen B, Shang X (2020). Predicting stage-specific recurrent aberrations from somatic copy number dataset. Front Genomics.

[6] Shi L, Tong W, Fang H, Scherf U, Han J, Puri RK, Frueh FW, Goodsaid FM, Guo L, Su Z (2005). Cross-platform comparability of microarray technology: intra-platform consistency and appropriate data analysis procedures are essential. BMC Bioinformatics.

[7] Kadota K (2011). Evaluating methods for ranking differentially expressed genes applied to microarray quality control data. BMC Bioinformatics.

[8] Lockhart DJ, Dong H, Byrne MC, Follettie MT, Gallo MV, Chee MS, Mittmann M, Wang C, Kobayashi M, Horton H (1996). Expression monitoring by hybridization to high-density oligonucleotide arrays. Nature Biotechnol.

[9] Mccarthy DJ, Smyth GK (2009). Testing significance relative to a fold-change threshold is a treat. Bioinformatics.

[10] Tusher VG, Tibshirani R, Chu G (2001). Significance analysis of microarrays applied to the ionizing radiation response. Proc Natl Acad Sci.

[11] Smyth GK (2004). Linear models and empirical bayes methods for assessing differential mir-483-5p identified as predictors of poor prognosis in adrenocortical cancer. Clin Cancer Res.

[12] Clark NR, Hu KS, Feldmann AS, Yan K, Chen EY, Duan Q, MaAyan A (2014). The characteristic direction: a geometrical approach to identify differentially expressed genes. BMC Bioinformatics.

[13] Chen B, Gao L, Shang X, Huang DS, Jo KH, Huang ZK (2019). Identifying Differentially Expressed Genes Based on Differentially Expressed Edges. Intelligent Computing Theories and Application. Lecture Notes in Computer Science, vol 11644.

[14] Liu JX, Xu Y, Gao YL, Zheng CH, Wang D, Zhu Q (2016). A class-information-based sparse component analysis method to identify differentially expressed genes on rna-seq data. IEEE/ACM Trans Comput Biol Bioinforma.

[15] Du J, Yang H, Tian D, Wang Q, He L (2014). Identification and functional analysis of differentially expressed genes related to obesity using dna microarray. Genet Mol Res.

[16] Silva GK, Costa RS, Silveira TN, Caetano BC, Horta CV, Gutierrez FR, Guedes PM, Andrade WA, De NM, Gazzinelli RT (2013). Apoptosis-associated speck-like protein containing a caspase recruitment domain inflammasomes mediate il-1b response and host resistance to trypanosoma cruzi infection. J Immunol.

[17] Bafica A, Santiago HC, Goldszmid R, Ropert C, Gazzinelli RT, Sher A. Cutting edge: Tlr9 and tlr2 signaling together account for myd88-dependent control of parasitemia in trypanosoma cruzi infection. J Immunol; 177(6):3515–9. 10.4049/jimmunol.177.6.3515 16951309

[18] Caetano B, Carmo B, Melo M, Cerny A, Santos S, Bartholomeu D, Golenbock D, Gazzinelli R (2011). Requirement of unc93b1 reveals a critical role for tlr7 in host resistance to primary infection with trypanosoma cruzi. J Immunol (Baltimore, Md. : 1950).

[19] Campos MA, Closel M, Valente EP, Cardoso JE, Akira S, Alvarez-Leite JI, Ropert C, Gazzinelli RT (2004). Impaired production of proinflammatory cytokines and host resistance to acute infection with Trypanosoma cruzi in mice lacking functional myeloid differentiation factor 88. J Immunol.

[20] Ricketts CJ, de Cubas AA, Fan H, Smith CC, Lang M, Reznik E, Bowlby R, Gibb EA, Akbani R, Beroukhim R, Bottaro DP, Choueiri TK, Gibbs RA, Godwin AK, Haake S, Hakimi AA, Henske EP, Hsieh JJ, Ho TH, Kanchi RS, Krishnan B, Kwaitkowski DJ, Lui W, Merino MJ, Mills GB, Myers J, Nickerson ML, Reuter VE, Schmidt LS (2018). The Cancer Genome Atlas Comprehensive Molecular Characterization of Renal Cell Carcinoma. Cell Rep.

[21] Silva GK, Gutierrez FRS, Guedes PMM, Horta CV, Cunha LD, Mineo TWP, Santiago-Silva J, Kobayashi KS, Flavell RA, Silva JS (2010). Cutting edge: Nucleotide-binding oligomerization domain 1-dependent responses account for murine resistance against trypanosoma cruzi infection. J Immunol.

[22] Cui HX, Liu RR, Zhao GP, Zheng MQ, Chen JL, Wen J (2012). Identification of differentially expressed genes and pathways for intramuscular fat deposition inpectoralis majortissues of fast-and slow-growing chickens. BMC Genomics.

[23] Zhu Q, Miecznikowski JC, Halfon MS. Preferred analysis methods for affymetrix genechips. ii. an expanded, balanced, wholly-defined spike-in dataset, Vol. 11; 2010. 10.1186/1471-2105-11-285 PMC2897828 20507584

[24] Dembélé D, Kastner P (2014). Fold change rank ordering statistics: a new method for detecting differentially expressed genes. BMC Bioinformatics.

[25] Pepper SD, Saunders EK, Edwards LE, Wilson CL, Miller CJ (2007). The utility of mas5 expression summary and detection call algorithms. BMC Bioinformatics.

[26] Xiao Y, Feng M, Ran H, Han X, Li X. Identification of key differentially expressed genes associated with non-small cell lung cancer by bioinformatics analyses, Vol. 17; 2018. 10.3892/mmr.2018.8726 PMC5928621 29532892

[27] Tang F, He Z, Lei H, Chen Y, Lu Z, Zeng G, Wang H. Identification of differentially expressed genes and biological pathways in bladder cancer, Vol. 17; 2018. 10.3892/mmr.2018.8711 PMC5928619 29532898

[28] De LRJ, Fontanillo C (2010). Protein-protein interactions essentials: key concepts to building and analyzing interactome networks. Plos Comput Biol.

[29] Christian VM, Jensen LJ, Berend S, Hooper SD, Markus K, Mathilde F, Nelly J, Huynen MA, Peer B (2005). String: known and predicted protein-protein associations, integrated and transferred across organisms. Nucleic Acids Res.

[30] Ogata H, Goto S, Sato K, Fujibuchi W, Bono H, Kanehisa M (2000). KEGG: Kyoto encyclopedia of genes and genomes. Nucleic Acids Res.

[31] Minoru K, Michihiro A, Susumu G, Masahiro H, Mika H, Masumi I, Toshiaki K, Shuichi K, Shujiro O, Toshiaki T (2007). KEGG for linking genomes to life and the environment. Nucleic Acids Res.

